# Sodium Concentration Measurement during Hemodialysis through Ion-Exchange Resin and Conductivity Measure Approach: In Vitro Experiments

**DOI:** 10.1371/journal.pone.0069227

**Published:** 2013-07-02

**Authors:** Andrea Tura, Stefano Sbrignadello, Emanuele Mambelli, Paolo Ravazzani, Antonio Santoro, Giovanni Pacini

**Affiliations:** 1 Institute of Biomedical Engineering, National Research Council, Padova, Italy; 2 Nephrology Dialysis and Hypertension Unit, Policlinico S. Orsola-Malpighi, Bologna, Italy; University of South Florida College of Medicine, United States of America

**Keywords:** sodium, dialysis, conductivity, resin, ion-exchange, ultrafiltration

## Abstract

Sodium measurement during hemodialysis treatment is important to preserve the patient from clinical events related to hypo- or hyper-natremia Usually, sodium measurement is performed through laboratory equipment which is typically expensive, and requires manual intervention. We propose a new method, based on conductivity measurement after treatment of dialysate solution through ion-exchange resin. To test this method, we performed in vitro experiments. We prepared 40 ml sodium chloride (NaCl) samples at 280, 140, 70, 35, 17.5, 8.75, 4.375 mEq/l, and some “mixed samples”, i.e., with added potassium chloride (KCl) at different concentrations (4.375-17.5 mEq/l), to simulate the confounding factors in a conductivity-based sodium measurement. We measured the conductivity of all samples. Afterwards, each sample was treated for 1 min with 1 g of Dowex G-26 resin, and conductivity was measured again. On average, the difference in the conductivity between mixed samples and corresponding pure NaCl samples (at the same NaCl concentration) was 20.9%. After treatment with the exchange resin, it was 14.7%, i.e., 42% lower. Similar experiments were performed with calcium chloride and magnesium chloride as confounding factors, with similar results. We also performed some experiments on actual dialysate solution during hemodialysis sessions in 15 patients, and found that the correlation between conductivity measures and sodium concentration improved after resin treatment (R=0.839 before treatment, R=0.924 after treatment, P<0.0001). We conclude that ion-exchange resin treatment coupled with conductivity measures may improve the measurement of sodium compared to conductivity measures alone, and may become a possible simple approach for continuous and automatic sodium measurement during hemodialysis.

## Introduction

The amount of sodium present in the body controls the extracellular volume. In advanced renal failure, sodium balance becomes positive and the extracellular volume expands, and this leads to adverse cardiovascular consequences in patients under hemodialysis (HD) treatment [1]. In fact, the sodium balance affects cardiovascular mortality of HD patients not only by raising blood pressure through increased extracellular volume, increased extracellular sodium content and increased total peripheral resistances [2], but also through an independent effect on heart hypertrophy and dilatation, on vascular smooth muscle cells’ hypertrophy and on reactive oxygen species promotion [3,4].

Thus, the maintenance of sodium balance is an essential item in chronic HD treatment. In HD, this balance is determined by salt intake during the interdialytic period and sodium removal in HD sessions. Most of the sodium in a HD session is removed by ultrafiltration of plasma water, and the diffusion process becomes responsible for the fine tuning of sodium balance [5]. As a consequence of a negative or positive sodium gradient between dialysate and plasma, patients can undergo an even greater removal of plasma sodium, or on the contrary may experience sodium gain [6]. In fact, with supraphysiologic dialysate sodium concentrations, diffusive influx from dialysate may occur, especially in patients with low predialytic plasma sodium concentrations, thus causing sodium load [7]. On the other hand, sodium removal should not be excessive, since this may lead to hypotensive events. Thus, reliable measurement of sodium concentration during HD is clinically relevant.

Currently, there are two main approaches for sodium measurement during HD: flame photometry or ion-selective electrode method, which includes both direct and indirect technology [8-10]. These methods are based on medical instruments, which in some cases also perform the measurement of several different variables (such as the hemogas-analyzers), but in any case they are not integrated with the HD machine. Thus, the measurement of sodium (and, possibly, other variables) always requires manual intervention by an operator, and this results in a severe drawback, especially in the case that the measurement is repeated several times during the HD session. In addition, the measurement performed by these instruments is typically very expensive.

The aim of this study is to propose an approach for a possible simple, not expensive, and automatic measurement of sodium during HD. Our approach is based on conductivity measurement coupled with the use of an ion-exchange resin. Conductivity measurement of plasma or dialysate sample alone cannot provide a highly accurate estimation of sodium concentration, though it should be recognized that the main contributor to conductivity of such samples is in fact sodium. However, coupling the simple conductivity measurement with treatment of the samples through an appropriate ion-exchange resin can lead to improved accuracy, since the effect of the disturbing factors (especially, other cations) is reduced. In this study, we focus on the presentation of our approach tested in artificial samples. However, some results are also reported on dialysate samples collected during HD sessions.

## Materials and Methods

### Preparation of samples

We prepared some artificial samples, at different concentration of sodium, potassium, calcium, magnesium, based on the following considerations. In normal physiological conditions, the sodium concentration in plasma is around 140 mEq/l; the concentration of the other cations, which represent the main confounders for conductivity-based sodium measurements, are around 4-5 mEq/l for potassium and calcium, and 1.5 mEq/l for magnesium [11,12].

Deionized water (18.5 MΩ × cm resistivity, Millipore MilliQ Element system, USA) and sodium chloride (NaCl, >99.5%, Sigma-Aldrich, Italy) was used to prepare a 500 ml sample of NaCl solution at 280 mEq/l, a concentration value certainly including the highest physiological plasma values (being twice the typical value). Afterwards, through dilution process, we obtained other six samples at lower concentrations, reaching the typical concentration values of plasma potassium and calcium: 140, 70, 35, 17.5, 8.75, 4.375 mEq/l (see Table 1).

**Table 1:** - Conductivity (mS/cm) of NaCl, KCl, CaCl_2_, MgCl_2_ samples at the different concentration (4.375-280 mEq/l), before and after treatment with the ion-exchange resin.

	Conductivity Pre-treatment/Post-treatment			
Concentration	NaCl	KCl	CaCl_2_	MgCl_2_
280	25.33/53.27	30.54/48.75	20.14/35.14	24.12/35.77
140	12.93/28.06	15.92/29.81	12.13/27.18	13.76/26.22
70	6.66/15.95	8.40/18.54	6.77/15.47	7.43/17.77
35	3.86/10.18	4.51/11.50	3.83/9.40	3.74/9.51
17.5	2.32/5.80	2.60/6.15	2.22/5.20	1.98/4.76
8.75	1.14/2.96	1.30/3.29	1.10/2.63	1.09/2.68
4.375	0.61/1.56	0.69/1.63	0.60/1.40	0.56/1.31

With the same process, with water and potassium chloride (KCl, >99.0%, Sigma-Aldrich), calcium chloride (CaCl_2_, >96.0%, Sigma-Aldrich), magnesium chloride (MgCl_2_, >98.0%, Sigma-Aldrich), we prepared samples at the same concentration of the sodium samples (i.e., from 280 to 4.375 mEq/l).

We also prepared some “mixed” samples, containing both sodium and potassium, in this case by direct weighting of the appropriate amount of NaCl and KCl for each sample. Specifically, we prepared a total of nine mixed samples: NaCl at 280 mEq/l and KCl at 17.5, 8.75, 4.375 mEq/l; NaCl at 140 mEq/l and KCl at 8.75, 4.375 mEq/l; NaCl at 70 mEq/l and KCl at 8.75, 4.375 mEq/l; NaCl at 35 mEq/l and KCl at 4.375 mEq/l; NaCl at 17.5 mEq/l and KCl at 4.375 mEq/l (see Table 2). Similar mixed samples were prepared with NaCl and CaCl_2_, and with NaCl and MgCl_2_ (see Table 3 and Table 4, respectively). Such mixed samples were prepared to simulate the presence of a confounding factor (i.e., potassium, calcium, and magnesium) for the measurement of sodium concentration in the sample. Of course, depending on the sodium concentration of the samples, we selected some significant but appropriately lower concentrations of the confounding factors, thus somehow mimicking what would happen in biological samples (see typical physiological plasma ion concentrations reported above). To further investigate the possible effect of such confounding factors, we also prepared a sample with 140 mEq/l of NaCl, and 4.375 mEq/l of KCl, CaCl_2_ and MgCl_2_. Finally, to this last sample we added 16.65 mmol/l (300 mg/dl) of glucose, i.e., a still physiological, but very high, glucose concentration value.

**Table 2:** Concentration of mixed samples (NaCl+KCl), and corresponding percentage difference in the conductivity (%) compared to the samples with NaCl alone at the same concentration, pre- and post-resin treatment (Δ_PRE_ and Δ_POST_, respectively).

NaCl (mEq/l)→	280	140	70	35	17.5
+					
KCl (mEq/l)					
↓					
Pre-treatment					
17.5	Δ_PRE_=17.28	‐	‐	‐	‐
8.75	Δ_PRE_=15.27	Δ_PRE_=19.93	Δ_PRE_=31.53	‐	‐
4.375	Δ_PRE_=12.78	Δ_PRE_=13.09	Δ_PRE_=20.54	Δ_PRE_=24.09	Δ_PRE_=33.13
Post-treatment					
17.5	Δ_POST_=11.55	‐	‐	‐	‐
8.75	Δ_POST_=11.17	Δ_POST_=13.00	Δ_POST_=20.64	‐	‐
4.375	Δ_POST_=7.62	Δ_POST_=5.81	Δ_POST_=18.55	Δ_POST_=23.46	Δ_POST_=20.26

**Table 3:** Concentration of mixed samples (NaCl+CaCl_2_), and corresponding percentage difference in the conductivity (%) compared to the samples with NaCl alone at the same concentration, pre- and post-resin treatment (Δ_PRE_ and Δ_POST_, respectively).

NaCl (mEq/l)→	280	140	70	35	17.5
+					
CaCl_2_ (mEq/l)					
↓					
Pre-treatment					
17.5	Δ_PRE_=12.32	‐	‐	‐	‐
8.75	Δ_PRE_=10.43	Δ_PRE_=39.71	Δ_PRE_=46.15	‐	‐
4.375	Δ_PRE_=2.36	Δ_PRE_=12.51	Δ_PRE_=36.79	Δ_PRE_=63.73	Δ_PRE_=30.49
Post-treatment					
17.5	Δ_POST_=6.05	‐	‐	‐	‐
8.75	Δ_POST_=4.95	Δ_POST_=4.74	Δ_POST_=37.70	‐	‐
4.375	Δ_POST_=2.62	Δ_POST_=3.48	Δ_POST_=19.44	Δ_POST_=37.98	Δ_POST_=31.05

**Table 4:** Concentration of mixed samples (NaCl+MgCl_2_), and corresponding percentage difference in the conductivity (%) compared to the samples with NaCl alone at the same concentration, pre- and post-resin treatment (Δ_PRE_ and Δ_POST_, respectively).

NaCl (mEq/l)→	280	140	70	35	17.5
+					
MgCl_2_ (mEq/l)					
↓					
Pre-treatment					
17.5	Δ_PRE_=21.83	‐	‐	‐	‐
8.75	Δ_PRE_=15.34	Δ_PRE_=15.17	Δ_PRE_=23.15	‐	‐
4.375	Δ_PRE_=10.39	Δ_PRE_=13.33	Δ_PRE_=26.37	Δ_PRE_=27.89	Δ_PRE_=17.79
Post-treatment					
17.5	Δ_POST_=5.88	‐	‐	‐	‐
8.75	Δ_POST_=5.03	Δ_POST_=9.38	Δ_POST_=14.86	‐	‐
4.375	Δ_POST_=2.23	Δ_POST_=9.24	Δ_POST_=16.99	Δ_POST_=12.91	Δ_POST_=12.06

### Conductivity measurement of the samples

We performed impedance measurement of the samples through a Solartron 1260 impedance analyzer (Solartron Analytical, UK), as already described in previous studies [13,14]. Through the Solartron 1260 we applied a 100 mV r.m.s. sinusoidal voltage to the outer couple of electrodes of the measurement probe (SP06T model, Delta OHM, Italy), which was immersed into the glass tube containing the analyzed sample. The electric current was measured through the inner electrodes of the probe. Separation of stimulation and sensing terminals allows minimization of possible secondary effects, such as inductance of cables or stray capacitances that can influence the accuracy of the impedance measurement [15]. We analyzed the impedance of the samples in the 10^3^–10^7^ Hz range. However, in this study we simply considered the impedance value at 1 kHz, as done in several commercial conductivity meters. To perform the transformation from impedance to conductivity, we used the formula C=1000*K/Z, where C is the conductivity (mS/cm), Z is the impedance (Ω) and K is the geometrical factor of the probe (k-factor), which is equal to 0.7 cm^-1^. For each measure, we used 40 ml of the solution sample of interest. For each sample studied, we performed two independent measurements: after the first measurement the cell was cleaned before immersing it again into the sample. The conductivity values presented for each sample are the average between the two measurements results, unless otherwise specified. All the impedance measurements were performed with the samples at ambient temperature (20 °C with maximum variations of ±0.3 °C).

In a first set of experiments, we measured the conductivity, as described above, in all the prepared samples. In a second set of experiments, we introduced the use of an ion-exchange resin, i.e., the Dowex G-26 exchange resin (Dow Chemical Company, USA). This is a uniform particle size, strong acid cation-exchange resin. It is composed by a polymeric matrix made of styrene-divinylbenzene, with a sulfonic acid as functional group. Such resin acts by replacing the cation species in a solution with hydrogen ions, H^+^. However, it should have a specially strong affinity with alkali metals, and with sodium in particular. Thus, we expected that the use of the resin determines variations in the characteristics of analyzed solution samples, related to the sodium concentration. In this second set of experiments, we immersed different quantities of resin in a 40 ml sample of NaCl at 140 mEq/l. Specifically, we tested the effect of 1, 2, 3, 4 g of resin. For each quantity of resin, we stirred the sample solution through an electromagnetic micro-stirrer (Velp Scientifica, Italy) for 1 min, in order to ensure optimal contact between the solution and the resin. Afterwards, the resin was separated by the sample solution, and conductivity measurement of the sample was performed. Subsequently, with 1 g of resin, we tested over the 140 mEq/l NaCl sample the effect of changing the contact time: in fact, we increased the time to 5, 10 and 20 min. We then repeated the experiments on different quantity of resin or contact time for the 35 mEq/l and 4.375 mEq/l NaCl samples.

Based on the results obtained in the second set of experiments, we proceeded with a third set of experiments. In this phase, each of the prepared samples, which underwent conductivity measurement in the first set of experiments, was treated with 1 g of resin for 1 min, and hence conductivity measurement was again performed.

### Analysis of dialysate samples

One sample of dialysate solution was collected during HD sessions in 15 patients included in this study. Patients provided written informed consent to the use of their data for clinical research purposes. The informed consent was approved by the S. Orsola-Malpighi Hospital (code RS02S, Rev. 0, application date 2001-08-01). All the patients’ data were collected anonymously; in addition, it should be noted that the collected samples were taken from the dialysate solution, generated by the HD machine through proper settings, before the contact with the patient’s blood. Thus, the approval of the ethics committee was not needed, as clarified in the document “Regolamento del Comitato Etico Indipendente dell’Azienda Ospedaliero - Universitaria di Bologna Policlinico S. Orsola-Malpighi”, approved by the ethics committee on February the 16th, 2010 (see specifically article 3.1 of that document).

After collection, samples were immediately analyzed with hemogas-analyzer GEM Premier 4000 (Instrumentation Laboratory, USA) for determination of ion concentrations (sodium, potassium, calcium, chlorine), and of other variables (glucose, lactate, oxygen and carbon dioxide partial pressure, and pH). Measurements were performed at 37 °C. After sample analysis, 40 ml of each sample were stored in a glass tube at -20 °C. Subsequently, each sample was taken back to ambient temperature, and underwent conductivity measurement, as already described. Similarly to the measurements with the artificial samples, the measurement of conductivity was repeated after treatment with 1 g of resin for 1 min.

### Statistical analyses

Linear regression analysis was performed to analyze the relationship between sodium, potassium, calcium and magnesium concentration and conductivity of the samples. Non-parametric Wilcoxon Signed Rank test was performed to assess possible differences in conductivity before and after the treatment with resin. The same test was used to assess the different effects of the resin on sodium, potassium, calcium and magnesium, and in the case of glucose presence. P<0.05 was considered as statistically significant. Data are reported as mean ±standard error.

## Results

The conductivity of the NaCl samples (with concentration from 280 to 4.375 mEq/l) is reported in Table 1. Conductivity increased linearly with the ion concentration. Similar trend was shown by the KCl, CaCl_2_, MgCl_2_ samples (Table 1).

In the analysis of the effect of the resin on the sample conductivity, we tested the conductivity variation of the NaCl sample at 140 mEq/l with 1, 2, 3 or 4 g of resin, with a contact time of 1 min. The treatment with the resin determined an increase in the conductivity of the sample. The conductivity difference (before and after resin treatment) was Δ=15.1 mS/cm with 1 g of resin. With increasing quantity of resin, the conductivity difference was Δ=20.9, Δ=24.8, Δ=26.3 mS/cm, for 2, 3, 4 g, respectively. Thus, with 1 g only, the effect of resin was 57% of that with 4 g. We also repeated these experiments on NaCl samples at different concentrations, and we found similar results: for the 35 mEq/l sample, the resin effect with 1 g was 69% of that with 4 g; for the 4.375 mEq/l sample, it was 53%. It can be concluded that, for the volume of our sample (40 ml), increasing the quantity of resin over 1 g determines an increase of the resin effect, but the effect is already clearly evident at 1 g. We also tested the changes in resin effect due to variations in the contact time between the resin and the sample. With the NaCl sample at 140 mEq/l, and with the use of 1 g of resin, we found Δ=16.8, Δ=19.4, Δ=20.1 mS/cm, for 5, 10, 20 min, respectively. Thus, with 1 min only, the effect of resin was 75% of that with 20 min; for the 35 mEq/l and 4.375 mEq/l samples, it was 71% and 55%, respectively. We concluded that the use of 1 g of resin, with a contact time of 1 min, provided an already marked resin effect, and it would therefore be sufficient for the treatment of our 40 ml samples.

The conductivity of the NaCl samples, and of the KCl, CaCl_2_, MgCl_2_ samples, treated with 1 g of resin for 1 min, are reported always in Table 1. At any concentration, the resin effect is clearly evident for all NaCl, KCl, CaCl_2_, MgCl_2_ samples. In fact, a significant average difference of the conductivity pre-post resin treatment was found for all NaCl, KCl, CaCl_2_, MgCl_2_ samples (P<0.02). However, for each concentration value it is possible to quantify the resin effect as the pre-post resin conductivity difference, normalized to the conductivity of the sample before resin treatment. For the NaCl samples, the resin effect was 1.43±0.08, whereas for the KCl, CaCl_2_, MgCl_2_ samples it was 1.21±0.13, 1.26±0.09, 1.22±0.14, respectively. Of note, the resin effect in NaCl was significantly higher than in KCl, CaCl_2_, MgCl_2_ (P<0.03 or lower). This confirms the stronger effect of the resin on sodium rather than on the other cations. Furthermore, it should be noted that the resin effect in KCl, CaCl_2_ and MgCl_2_ was similar (P>0.7).

We then analyzed the mixed samples, containing NaCl and KCl, or NaCl and CaCl_2,_ or NaCl and MgCl_2_, to simulate the presence of a confounding factor (i.e., potassium, or calcium, or magnesium) for the measurement of sodium concentration of the sample. Conductivity measurement of each sample was performed, and then repeated after treatment with the resin (1 g for 1 min, as for the previously analyzed samples). For each mixed sample, we computed the percentage difference in conductivity between the mixed sample and the corresponding pure NaCl sample, at the same NaCl concentration (for instance, the conductivity difference between the 280 mEq/l NaCl + 17.5 mEq/l KCl sample and the pure 280 mEq/l NaCl sample, normalized to the latter.). We computed such difference before and after the treatment with the resin. Pre- and post-treatment conductivity difference is reported in Table 2 for NaCl and KCl, in Table 3 for NaCl and CaCl_2,_ and in Table 4 for NaCl and MgCl_2_. It can be noticed that, for almost all the mixed samples, the difference in conductivity with the corresponding pure sample was lower after the treatment with the resin. On average, in NaCl and KCl the conductivity difference before resin treatment was 20.9%; after treatment, it was 14.7%, i.e., 42% lower; in NaCl and CaCl_2_ it was 28.3% and 16.5% (72% lower), and in NaCl and MgCl_2,_ it was 19.0% and 9.8% (93% lower). As regards the sample with the presence of all the cations (140 mEq/l NaCl, 4.375 KCl, CaCl_2_ and MgCl_2_), the difference in conductivity with the corresponding pure sample (140 mEq/l NaCl alone) was 15.7% before resin treatment, and 8.3% after treatment (i.e., 89% lower). All these findings show that, in the analyzed artificial samples, the treatment with resin reduced the confounding effect of other cations on the assessment of sodium concentration, based on conductivity measurement approach. In the sample with all cations, we also added glucose at very high physiological concentration value. We performed conductivity measurement, then resin treatment and subsequent new conductivity measurement, for 10 times; we then computed the resin effect (pre-post resin conductivity difference normalized to pre-resin conductivity), and obtained 1.03±0.03. We compared this result with the resin effect of the sample with all cations but no glucose, for which the conductivity measure was similarly repeated for 10 times (resin effect equal to 1.14±0.08), and found that the difference was not significant (P>0.2). Thus, the presence of glucose, even at high concentration values, does not significantly influence the effect of the resin on the sample.

**Figure 1:**
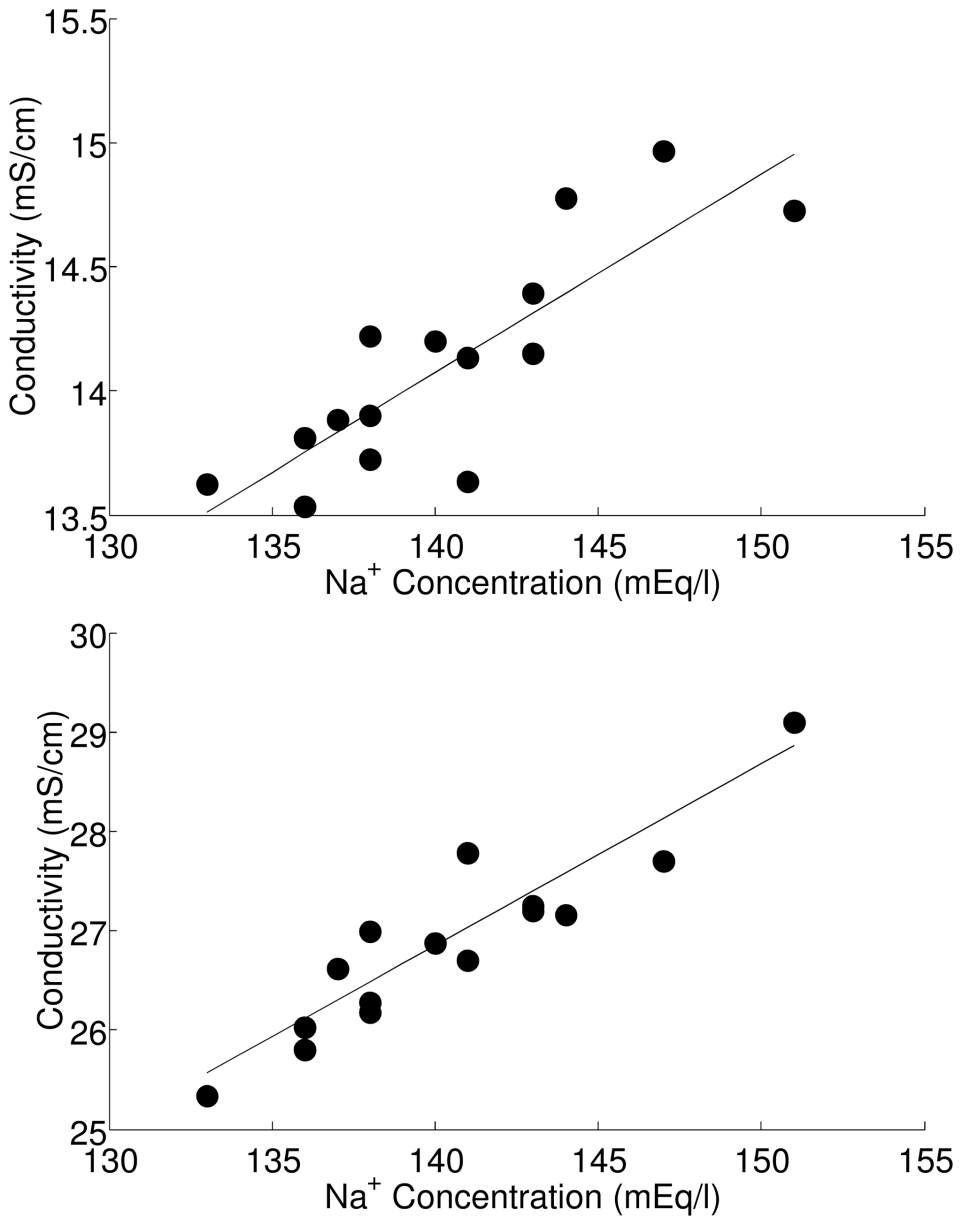
Conductivity of the dialysate solution samples in relation to sodium concentration. Conductivity is reported before (top) and after (bottom) treatment with ion-exchange resin. R was 0.839 and 0.924, respectively, P<0.0001. Regression equation was y = 0.08x+2.87 and y=0.18x+1.23.

We also analyzed some samples of dialysate solution collected during HD session. The main variables that were measured in these samples are reported in Table 5. Relationship between the sodium concentration and the conductivity of the sample is reported in Figure 1, top panel: a good correlation was found (R=0.839, P<0.0001). However, after treatment with the resin (again 1 g for 1 min) the correlation was even slightly higher (R=0.924, P<0.0001; Figure 1, bottom panel).

**Table 5:** Concentration of ions and other variables in dialysate solution samples of fifteen subjects during hemodialysis: sodium (Na^+^), potassium (K^+^), calcium (Ca^2+^), chlorine (Cl^-^), glucose (Glu), lactate (Lact), oxygen partial pressure (pO_2_), carbon dioxide partial pressure (pCO_2_), pH.

	Na^+^	K^+^	Ca^2+^	Cl^-^	Glu	Lact	pO_2_	pCO_2_	pH
	(mEq/l)	(mEq/l)	(mEq/l)	(mEq/l)	(mg/dl)	(mmol/l)	(mmHg)	(mmHg)	(non-dim)
Subject_1	136	2.8	2.68	111	94	<0.3	138	47	7.47
Subject_2	143	3.1	2.72	122	104	<0.3	216	32	7.62
Subject_3	141	3	2.72	120	102	<0.3	193	32	7.61
Subject_4	141	2.8	2.62	117	95	<0.3	29	7.66
Subject_5	138	2.9	2.58	116	98	<0.3	198	30	7.67
Subject_6	151	3.1	2.82	127	102	<0.3	194	31	7.61
Subject_7	138	2.9	2.36	117	100	<0.3	197	22	7.81
Subject_8	138	2.9	2.34	115	96	<0.3	190	18	7.85
Subject_9	143	3.1	2.68	121	103	<0.3	197	32	7.64
Subject_10	144	3.1	2.78	122	104	<0.3	192	34	7.61
Subject_11	133	2.7	2.54	109	93	<0.3	133	45	7.5
Subject_12	140	3	2.82	113	99	<0.3	135	60	7.32
Subject_13	137	3.1	2.44	112	105	0.4	154	31	7.6
Subject_14	136	2.8	2.64	111	93	<0.3	145	42	7.52
Subject_15	147	3.1	2.86	121	101	<0.3	122	77	7.28

## Discussion

In this study we aimed at proposing an approach that may contribute to overcome the current limitations in the measurement of sodium during HD. In fact, flame photometry and direct or indirect potentiometry are the most common techniques to measure sodium during HD [8-10]. Although these techniques usually provide results sufficiently accurate for the clinical purposes, possible problems have been reported. In fact, flame photometry measures the ion concentration, whereas potentiometry measures ion activity. Therefore, differences in the measurements by the two approaches have emerged, unless proper correction factors and calibration procedures are taken into account [16]. Furthermore, these methods are based on medical instruments that are not integrated within the HD machine. Thus, currently the measurement of sodium always requires manual intervention by an operator, and hence it is extremely time-consuming, especially when the measurement is repeated several times during the HD session. Moreover, the measurement performed by these instruments is quite expensive: for instance, a 75-test cartridge for hemogas-analyzer, for the measurement of sodium and other variables, can cost around eight hundred euros.

Our approach is based on conductivity measurement of plasma or dialysate. The measurement of conductivity during hemodialysis has been proposed in previous studies [17-21]. In the study [17], conductivity of dialysate was measured at the dialyzer inlet and outlet for two different dialysate conductivity values, and through a two equation model the patient’s conductivity and the dialysance of the dialyzer were calculated. In the study [18], the dialysance of the dialyzer, calculated with the same conductivity-based approach of the study [17], was used to assess the urea clearance, and hence the plasma volume cleared of urea. However, these two studies did not report information about relationship between dialysate conductivity (or patient’s conductivity) and sodium concentration. Such information was instead reported in the studies [19-21]. In [19], R=0.87, P<0.001 was found for the correlation between patient’s conductivity and plasma sodium concentration in 133 samples from 64 patients. In the studies [20-21], even higher correlations (R=0.922-0.95) were found between plasma conductivity and sodium concentration, though on a lower number of samples (38 and 24, from 17 and 12 patients). More recently, conductivity measurement of ultrafiltrate for sodium determination has been proposed by the company Bellco, Italy (http://www.bellco.net/public/files/Right_Therapies_News.pdf. Last checked: 7/12/2012). Though in the studies [19-21] the correlation between conductivity and sodium concentration appears quite strong, simple conductivity measurement cannot completely guarantee highly accurate estimation of sodium concentration, due to the possible influence of several disturbing factors: namely, the other cations (potassium, calcium, magnesium), but also glucose, which proved to affect the impedance of a solution, though slightly, despite the fact that it does not decompose into ions [13, 14, 22-24].

The main novelty of this study is the idea of coupling the simple conductivity measurement with treatment of the solution sample with a not expensive (~ point zero four euro per gram) acid cation-exchange resin, which can reduce the effect of the disturbing factors in sodium determination. In fact, the contact of the solution with the resin determines an “amplification” of the sample conductivity, which depends on the concentration of the cations that are present, and, especially, the alkali metals. The resin also reacts to the presence of alkaline earth metals (calcium and magnesium, in our case), but the affinity is expected to be lower with respect to the alkali metals (sodium and potassium). In fact, we have verified that the resin effect on calcium and magnesium was lower than the effect on sodium. As regards potassium, though both sodium and potassium are alkali metals, we have shown that the resin effect is stronger on sodium. This may be due to the fact that potassium is less electronegative than sodium. Based on these considerations, it can be claimed that treating a sample with the resin determines an effect on conductivity which is mainly in relation to the sodium concentration. As a consequence, in the conductivity-based determination of the sodium concentration of the sample, resin treatment allows reducing the effect of the disturbing factors. In fact, in our artificial mixed samples (i.e., with sodium, potassium, calcium and magnesium at different concentrations), we found that the error in the conductivity-based sodium determination was reduced with resin treatment: 14.7%, instead of 20.9% without resin treatment, in the case of potassium as the disturbing factor. Similar results were found when the disturbing factor was calcium or magnesium, or when the disturbing factors were considered all together. Of note, as expected we have also found that glucose does not affect the resin behavior: in fact, glucose in water does not provide cations, thus it does not contribute to the cation-exchange process due to the presence of resin.

In the analysis of the actual dialysate samples, the advantage in the use of the resin was less evident: however, Figure 1 suggests that the correlation between conductivity and sodium concentration was improved after resin treatment, as mirrored by the slightly higher R value (0.924 vs. 0.839). It should be noted that the correlation coefficient found without resin treatment was similar to that of the study [19], where R was 0.87. Notably, the regression equations were also very similar (y=0.08x+2.87 in our study and y=0.08x+3.08 in the study [19]). As regards studies [20-21], the correlation coefficient was even higher (0.922 and 0.95): a possible explanation may be that, for some reasons, the concentration of the confounding factors (mainly, the other cations) was lower in the samples of studies [20-21] than in our samples. Unfortunately, this cannot be verified, since in studies [20-21] no information was reported about other cations, differently from our study. In addition, in all three studies [19-21], more than one sample was taken from the same patient, and hence the hypothesis that all observations are independent of each other, which should apply to regression analysis, may be not satisfied, thus possibly influencing the regression results.

In our resin-based method, a slightly different approach may be to consider the difference in conductivity before and after the treatment with resin, rather than the conductivity measure after resin treatment, as we have proposed in this study. However, this implies having to carry out two conductivity measurements rather than one, and the total error may be higher. In fact, we performed some calculations on our samples and did not find relevant advantages with such double measurement approach (not shown). In addition, it would be slightly more complex for the possible implementation on a HD machine. For these reasons, the single conductivity measure after resin treatment may be the most convenient choice.

A possible approach for future implementation of our method on a HD machine would require insertion of a measurement flow bypass on the ultrafiltration line. Some dialysate solution would be diverted and directed through the cartridge containing the ion-exchange resin. After the resin treatment, the dialysate solution would reach a simple two electrodes sensor for the conductivity measurement, and finally a probe would collect the measured solution, which would be eventually discarded as waste, together with the spent dialysate solution. In the case of treatment of a plasma sample, the need for discarding the treated sample is due to the fact that the resin action determines changes in the sample chemical composition: in particular, the alkali/alkaline-earth metal cations (especially sodium) are removed, and hydrogen cations released, with consequent pH change; thus the sample could not be safely reinfused to the patient. However, this is not a significant limitation, since the quantity of solution to be treated with resin for our purposes would be extremely small. By proper design of the described conceptual technical scheme, it would be possible to automatically repeat the measure several time during the HD session: for instance, an appropriate quantity of resin should be inserted in the cartridge to ensure optimal resin performance in all the measures planned during the HD session.

The study has two main limitations. First, in this study we used, for simplicity, dialysate samples taken before the dialyzer; however, in a possible implementation of the technique on a HD machine, dialysate samples must be collected and treated after the dialyzer (i.e., following the contact with the patient’s plasma). The second limitation is the low number of dialysate samples that we studied. In fact, this study was mainly devoted to the presentation of our approach for possible sodium measurement, and the analysis of the resin performances. Future studies should analyze samples collected from both chronic and acute patients in course of different dialysis techniques (Bicarbonate, Hemodiafiltration, AcetateFree Biofiltration, etc.).

In conclusion, we have proposed an approach for simple, automatic and inexpensive measurement of sodium. The approach is based on the use of a ion-exchange resin coupled with simple two electrodes conductivity measurement of samples of dialysate solution. Our results showed that the accuracy in the conductivity-based measurement of sodium can be improved. Further studies are necessary to fully prove the actual performance of the proposed approach, and test the possibility of integrating it on a HD machine.

## References

[1] Charra B (2007) Fluid balance, dry weight, and blood pressure in dialysis. Hemodial Int 11: 21–31. doi:10.1111/j.1542-4758.2007.00198.x. PubMed: 17257351. 17257351 10.1111/j.1542-4758.2007.00148.x

[2] Guyton AC, Coleman TG (1999) Quantitative analysis of the pathophysiology of hypertension. 1969. J Am Soc Nephrol 10: 2248–2258. PubMed: 10505704. 10505704

[3] Titze J, Krause H, Hecht H, Dietsch P, Rittweger J et al. (2002) Reduced osmotically inactive Na storage capacity and hypertension in the Dahl model. Am J Physiol Renal Physiol 283: F134-F141. PubMed: 12060595. 12060595 10.1152/ajprenal.00323.2001

[4] Kitiyakara C, Chabrashvili T, Chen Y, Blau J, Karber A et al. (2003) Salt intake, oxidative stress, and renal expression of NADPH oxidase and superoxide dismutase. J Am Soc Nephrol 14: 2775-2782. doi:10.1097/01.ASN.0000092145.90389.65. PubMed: 14569087. 14569087 10.1097/01.asn.0000092145.90389.65

[5] Santos SF, Peixoto AJ (2008) Revisiting the dialysate sodium prescription as a tool for better blood pressure and interdialytic weight gain management in hemodialysis patients. Clin J Am Soc Nephrol 3: 522–530. doi:10.2215/CJN.03360807. PubMed: 18199846. 18199846 10.2215/CJN.03360807 PMC6631087

[6] Hecking M, Kainz A, Hörl WH, Herkner H, Sunder-Plassmann G (2011) Sodium setpoint and sodium gradient: influence on plasma sodium change and weight gain. Am J Nephrol 33: 39-48. doi:10.1159/000322572. PubMed: 21160174. 21160174 10.1159/000322572

[7] Kim GH (2009) Dialysis unphysiology and sodium balance. Electrolyte Blood Press 7:31-7. 10.5049/EBP.2009.7.2.31 PMC3041490 21468183

[8] Maas AH, Siggaard-Andersen O, Weisberg HF, Zijlstra WG (1985) Ion-selective electrodes for sodium and potassium: a new problem of what is measured and what should be reported. Clin Chem 31: 482-485. PubMed: 3971572. 3971572

[9] Worth HG (1985) A comparison of the measurement of sodium and potassium by flame photometry and ion-selective electrode. Ann Clin Biochem 22 ( Pt 4): 343-350. PubMed : 3898973 10.1177/000456328502200402 3898973

[10] Burnett RW, Covington AK, Fogh-Andersen N, Külpmann WR, Lewenstam A et al. (2000) Recommendations for measurement of and conventions for reporting sodium and potassium by ion-selective electrodes in undiluted serum, plasma or whole blood. International Federation of Clinical Chemistry and Laboratory Medicine(IFCC). IFCC Scientific Division Working Group on Selective Electrodes. Clin Chem Lab Med 38:1065-71 10.1515/CCLM.2000.159 11140625

[11] Caduff A, Lutz HU, Heinemann L, Di Benedetto G, Talary MS et al. (2011) Dynamics of blood electrolytes in repeated hyper- and/or hypoglycaemic events in patients with type 1 diabetes. Diabetologia 54: 2678-2689. doi:10.1007/s00125-011-2210-9. PubMed: 21674178. 21674178 10.1007/s00125-011-2210-9

[12] Musso CG (2009) Magnesium metabolism in health and disease. Int Urol Nephrol 41: 357-362. doi:10.1007/s11255-009-9548-7. PubMed: 19274487. 19274487 10.1007/s11255-009-9548-7

[13] Tura A, Sbrignadello S, Barison S, Conti S, Pacini G (2007) Impedance spectroscopy of solutions at physiological glucose concentrations. Biophys Chem 129: 235-241. doi:10.1016/j.bpc.2007.06.001. PubMed: 17602824. 17602824 10.1016/j.bpc.2007.06.001

[14] Sbrignadello S, Tura A, Ravazzani P (2013) Electroimpedance spectroscopy for the measurement of the dielectric properties of sodium chloride solutions at different glucose concentrations. J of SPECTROSCOPY, Article ID: 571372, pp. 6

[15] Awan SA, Kibble BP (2005) Towards accurate measurement of the frequency dependence of capacitance and resistance standards up to 10 MHz. IEEE Trans Instrum Meas 54: 516-520. doi:10.1109/TIM.2005.843582.

[16] Ladenson JH (1977) Direct potentiometric analysis of sodium and potassium in human plasma: evidence for electrolyte interaction with a nonprotein, protein-associated substance(s). J Lab Clin Med 90: 654-665. PubMed: 20480. 20480

[17] Petitclerc T, Goux N, Reynier AL, Béné B (1993) A model for non-invasive estimation of in vivo dialyzer performances and patient’s conductivity during hemodialysis. Int J Artif Organs 16: 585-591. PubMed: 8225649. 8225649

[18] Petitclerc T, Béné B, Jacobs C, Jaudon MC, Goux N (1995) Non-invasive monitoring of effective dialysis dose delivered to the haemodialysis patient. Nephrol Dial Transplant 10: 212-216. PubMed: 7753455. 7753455

[19] Petitclerc T, Hamani A, Jacobs C (1992) Optimization of Sodium Balance during Hemodialysis by Routine Implementation of Kinetic Modeling - Technical Aspects and Preliminary Clinical Study. Blood Purif 10: 309-316. doi:10.1159/000170062.

[20] Goureau Y, Petitclerc T, Man NK (1990) Evaluation of plasma sodium concentration during hemodialysis by computerization of dialysate conductivity. ASAIO Trans 36: M444-M447. PubMed: 2252723. 2252723

[21] Locatelli F, Di Filippo S, Manzoni C, Corti M, Andrulli S et al. (1995) Monitoring sodium removal and delivered dialysis by conductivity. Int J Artif Organs 18: 716-721. PubMed: 8964634. 8964634

[22] Gourzi M, Rouane A, Guelaz R, Nadi M, Jaspard F (2003) Study of a new electromagnetic sensor for glycaemia measurement: in vitro results on blood pig. J Med Eng Technol 27: 276-281. doi:10.1080/0309190031000098845. PubMed: 14602519. 14602519 10.1080/0309190031000098845

[23] Gourzi M, Rouane A, Guelaz R, Alavi MS, McHugh MB et al. (2005) Non-invasive glycaemia blood measurements by electromagnetic sensor: study in static and dynamic blood circulation. J Med Eng Technol 29: 22-26. doi:10.1080/03091900410001720247. PubMed: 15764378. 15764378 10.1080/03091900410001720247

[24] Tura A, Sbrignadello S, Cianciavicchia D, Pacini G, Ravazzani P (2010) A low frequency electromagnetic sensor for indirect measurement of glucose concentration: in vitro experiments in different conductive solutions. Sensors (Basel) 10: 5346-5358. doi:10.3390/s100605346. PubMed: 22219665. 22219665 10.3390/s100605346 PMC3247710

